# Increased *Ifi202b/IFI16* expression stimulates adipogenesis in mice and humans

**DOI:** 10.1007/s00125-018-4571-9

**Published:** 2018-02-24

**Authors:** Mandy Stadion, Kristin Schwerbel, Antonia Graja, Christian Baumeier, Maria Rödiger, Wenke Jonas, Christian Wolfrum, Harald Staiger, Andreas Fritsche, Hans-Ulrich Häring, Nora Klöting, Matthias Blüher, Pamela Fischer-Posovszky, Tim J. Schulz, Hans-Georg Joost, Heike Vogel, Annette Schürmann

**Affiliations:** 1 Department of Experimental Diabetology, German Institute of Human Nutrition Potsdam-Rehbruecke (DIfE), Arthur-Scheunert-Allee 114–116, D-14558 Nuthetal, Germany; 2 German Center for Diabetes Research (DZD), Munich, Neuherberg Germany; 3 Department of Adipocyte Development and Nutrition, German Institute of Human Nutrition Potsdam-Rehbruecke (DIfE), Nuthetal, Germany; 4 Institute of Food, Nutrition and Health, ETH Zürich, Schwerzenbach, Switzerland; 5 Institute of Pharmaceutical Sciences, Eberhard Karls University Tübingen, Tübingen, Germany; 6 Division of Endocrinology, Diabetology, Angiology, Nephrology and Clinical Chemistry, Department of Internal Medicine, University Hospital Tübingen, Tübingen, Germany; 7 IFB AdiposityDiseases, University of Leipzig, Leipzig, Germany; 8 Department of Medicine, University of Leipzig, Leipzig, Germany; 9 Division of Pediatric Endocrinology and Diabetes, Department of Pediatrics and Adolescent Medicine, University Medical Center Ulm, Ulm, Germany

**Keywords:** Adipogenesis, Beiging, *IFI16*, *Ifi202b*, Obesity, *Zfp423*

## Abstract

**Aims/hypothesis:**

Obesity results from a constant and complex interplay between environmental stimuli and predisposing genes. Recently, we identified the IFN-activated gene *Ifi202b* as the most likely gene responsible for the obesity quantitative trait locus *Nob3* (New Zealand Obese [NZO] obesity 3). The aim of this study was to evaluate the effects of *Ifi202b* on body weight and adipose tissue biology, and to clarify the functional role of its human orthologue *IFI16*.

**Methods:**

The impact of *Ifi202b* and its human orthologue *IFI16* on adipogenesis was investigated by modulating their respective expression in murine 3T3-L1 and human Simpson-Golabi-Behmel syndrome (SGBS) pre-adipocytes. Furthermore, transgenic mice overexpressing IFI202b were generated and characterised with respect to metabolic traits. In humans, expression levels of *IFI16* in adipose tissue were correlated with several variables of adipocyte function.

**Results:**

In mice, IFI202b overexpression caused obesity (Δ body weight at the age of 30 weeks: 10.2 ± 1.9 g vs wild-type mice) marked by hypertrophic fat mass expansion, increased expression of *Zfp423* (encoding the transcription factor zinc finger protein [ZFP] 423) and white-selective genes (*Tcf21*, *Tle3*), and decreased expression of thermogenic genes (e.g. *Cidea*, *Ucp1*). Compared with their wild-type littermates, *Ifi202b* transgenic mice displayed lower body temperature, hepatosteatosis and systemic insulin resistance. Suppression of IFI202b/IFI16 in pre-adipocytes impaired adipocyte differentiation and triacylglycerol storage. Humans with high levels of *IFI16* exhibited larger adipocytes, an enhanced inflammatory state and impaired insulin-stimulated glucose uptake in white adipose tissue.

**Conclusions/interpretation:**

Our findings reveal novel functions of *Ifi202b* and *IFI16*, demonstrating their role as obesity genes. These genes promote white adipogenesis and fat storage, thereby facilitating the development of obesity-associated insulin resistance.

**Electronic supplementary material:**

The online version of this article (10.1007/s00125-018-4571-9) contains peer-reviewed but unedited supplementary material, which is available to authorised users.

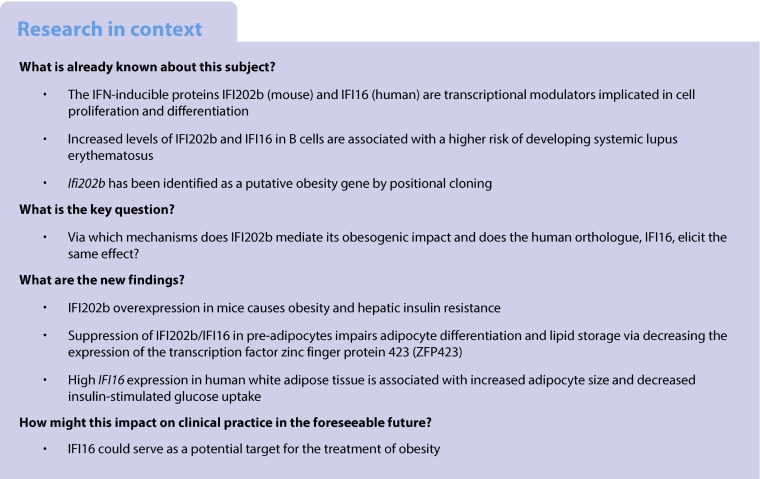



## Introduction

Obesity, an excessive expansion of adipose tissue, associates with metabolic perturbations that increase the risk for non-alcoholic fatty liver disease, type 2 diabetes and coronary heart disease. The increase in adipocyte size (hypertrophy) and adipocyte number (hyperplasia) both contribute to the development of obesity [1]. Prolonged overnutrition and low levels of physical activity result in adipose tissue dysfunction, characterised by adipocyte hypertrophy, macrophage infiltration, impaired insulin signalling and insulin resistance [2]. In addition, inflammatory cytokine levels rise and, together with excessive amounts of NEFA, promote ectopic fat deposition and lipotoxicity in the liver, muscle and pancreas. These effects ultimately contribute to the development of insulin resistance [3, 4]. Two types of adipose tissue can be distinguished: white adipose tissue (WAT), which stores excess energy as triacylglycerol and brown adipose tissue (BAT), which is specialised in the dissipation of energy through the production of heat [5]. Relatively high quantities of BAT are associated with lower body weight and BAT decreases with age [6]. In addition, a third type of fat cell, referred to as beige or brite (brown in white), has been recognised in certain kinds of WAT depots [7, 8].

Adipocyte commitment and differentiation are complex processes tightly controlled by a transcriptional cascade composed of several transcription factors, among which zinc finger protein (ZFP) 423, CCAAT/enhancer-binding proteins (C/EBPs) and Kruppel-like factors (e.g. KLF15) play an essential role [9, 10]. In 3T3-L1 murine pre-adipocytes, C/EBPβ is induced early in the differentiation process to activate the expression of C/EBPα and peroxisome proliferator-activated receptor γ (PPARγ), the two master regulators of terminal adipocyte differentiation [11, 12].

Previously, to identify obesity and diabetes genes, we performed linkage studies in an intercross of lean C57BL/6J and obese New Zealand Obese (NZO) mice resulting in a major obesity quantitative trait locus (QTL), *Nob3* (NZO obesity 3), on chromosome 1 [13]. By positional cloning, we defined *Ifi202b* (IFN-activated gene 202B) as a putative obesity gene [14]. We further showed that *Ifi202b* plays a role as a diabetes gene as its overexpression in primary islet cells inhibited proliferation [15]. IFN-inducible protein IFI202b and the human orthologue IFI16 belong to the p200 family of transcriptional modulators of cell proliferation, differentiation, apoptosis and inflammation [16, 17].

The aim of the current work was to provide direct functional evidence for *Ifi202b* as an obesity gene and to clarify the role of its human orthologue *IFI16* in obesity-associated insulin resistance. To study the impact of *Ifi202b* on adipose tissue biology and to investigate the underlying mechanisms of its obesogenic potency, we overexpressed IFI202b in 3T3-L1 pre-adipocytes and in lean C57BL/6J mice lacking endogenous *Ifi202b* because of a vast deletion upstream of exon 2 [18]. The effects of the human orthologue *IFI16* were analysed in Simpson–Golabi–Behmel syndrome (SGBS) pre-adipocytes, as well as in obese human volunteers.

## Methods

### Animals

C57BL/6J mice overexpressing IFI202b, herein referred to as B6-Tg(*Ifi202b*) mice, were generated by Ozgene (Perth, WA, Australia). c-Myc-tagged *Ifi202b* cDNA downstream of the human ubiquitin C promoter was integrated into the ROSA26 locus. C57BL/6J wild-type (B6-wt) littermates were used as control animals. NZO mice were taken from our own colony (NZO/HIBomDife, Nuthetal, Germany). For details of housing and diet conditions, see electronic supplementary materials (ESM) Methods.

### Characterisation of mice

Blood glucose, plasma variables (insulin, NEFA and triacylglycerol levels), glucose tolerance and body composition were assessed as described previously [18]. Plasma adiponectin and leptin levels were measured using the Mouse Adiponectin/Acrp30 and Mouse/Rat Leptin ELISA (DY1119 and MOB00, respectively; R&D Systems, Minneapolis, MN, USA). Histological analysis of WAT, BAT and liver tissues was carried out using H&E staining (see ESM Methods for further details).

### Intraperitoneal ITT and insulin sensitivity

For the i.p. ITT, 12- and 20-week-old non-fasted mice were injected with insulin (0.75 IU/kg body weight; Actrapid Penfill; Novo Nordisk, Mainz, Germany) and blood glucose levels were measured at various time points. To examine hepatic and muscle insulin sensitivity, after a 6 h fast, mice were injected with NaCl (control) or insulin (0.75 IU/kg body weight). Twenty minutes later, mice were killed by cervical dislocation under isoflurane anaesthesia and samples were taken for western blot analysis.

### Ex vivo lipolysis assay

Gonadal WAT (WATgon) explants from 8-week-old mice were stimulated with 10 μmol/l isoprenaline (known as isoproterenol in the USA) (Sigma-Aldrich, St Louis, MO, USA) or isoprenaline plus insulin (178 μmol/l) in 160 μl of high-glucose DMEM (PAN-Biotech, Aidenbach, Germany). After 2 h, release of NEFA was detected (NEFA-HR(2); Wako Chemicals, Neuss, Germany).

### Cell culture and differentiation

For details of murine 3T3-L1 and human SGBS cell culture and differentiation conditions, see ESM Methods. WT-1 brown pre-adipocytes [19] tested negative for mycoplasma and cultivated and differentiated as described [20], with modified dexamethasone concentration (5 μmol/l).

### Overexpression of IFI202b and analysis of lipid droplet formation in 3T3-L1 pre-adipocytes

3T3-L1 pre-adipocytes were infected with a c-Myc-*Ifi202b* encoding adenovirus (ViraQuest, North Liberty, IA, USA). Empty adenovirus was used as a control. Lipid droplet formation in IFI202b-overexpressing 3T3-L1 adipocytes was assessed by fluorescent staining. A detailed protocol for IFI202b overexpression in pre-adipocytes can be found in ESM Methods.

### siRNA-mediated knockdown of IFI202B/IFI16 in pre-adipocytes

Expression of IFI202b in murine 3T3-L1 and WT-1 pre-adipocytes, as well as IFI16 in human pre-adipocytes was suppressed via electroporation using specific siRNA (IFI16: J-020004-05-0050; Dharmacon, Lafayette, CO, USA) as described previously [14].

### Isolation and differentiation of primary adipogenic precursor cells

Adipogenic precursor cells were isolated from subcutaneous WAT (WATsc) of 8-week-old mice fed a standard diet (V153x R/M-H; ssniff, Soest, Germany), as described [21]. Stem cell antigen 1 (SCA1)-positive cells [22, 23] were purified by FACS (BD FACSAria III; BD Biosciences, San Jose, CA, USA) and differentiated, as previously described [21], with minor modifications.

### RNA extraction and expression analysis

RNA from cells was isolated and reverse transcribed for quantitative real-time PCR analyses. Expression levels of *Ifi202b*, *Zfp423*, *Pparg*, *Cebpa*, *Adipoq*, *Glut4* (also known as *Slc2a4*), *Fabp4*, *Plin1*, *Hsd11b1*, *Pref1* (*Dlk1*), *Ucp1*, *Cidea*, *Prdm16*, *Tcf21*, *Tle3*, *Ebf2*, *Ppargc1a*, *Ppara*, *Il4*, *Il6*, *Il10*, *Tnfa* (*Tnf*) and *Ccl2* were analysed in murine samples. In human samples, *IFI16*, *ZNF423*, *CEBPA*, *PPARG*, *ADIPOQ*, *FASN*, *GLUT4* (*SLC2A4*), *PLIN1* and *IL6* were analysed. Genes were detected using specific TaqMan Gene Expression Assays and the 7500 Fast Real-Time PCR System (Thermo Fisher Scientific, Waltham, MA, USA) and gene expression profiling of WATgon of 8-week-old mice was performed by OakLabs (Henningsdorf, Germany). For details see ESM Methods.

### Protein extraction and western blotting

Liver, muscle, BAT and WAT tissues from mice were homogenised and analysed by western blotting [24]. A monoclonal anti-IFI202b antibody was raised against the C-terminal peptide 1–21: C-MSNRNLRSSTNSEFSEGQHQ. Primary antibodies against c-Myc (1:500 dilution; Clontech, Saint-Germain-en-Laye, France), uncoupling protein 1 (UCP1; 1:1000; ab10983; Abcam, Cambridge, UK), total Akt (t-Akt; 1:1000; no. 9272; Cell Signaling, Danvers, CO, USA), p-Akt (Ser473; 1:1000; no. 9271; Cell Signaling), HDAC1 (1:25,000; ab7028; Abcam) and GAPDH (1:20,000; AM4300; Thermo Fisher Scientific), and appropriate horseradish peroxidase-labelled secondary antibodies (Dianova, Hamburg, Germany) were applied. Experimental controls were used to validate antibodies.

### *IFI16* expression in human adipose tissue

Previously, *IFI16* expression in adipose tissue was measured in 166 obese individuals [14]. Here, we compared those exhibiting the 10% highest levels of *IFI16* with those with the 10% lowest levels of expression. Human phenotyping [25], *IFI16* expression analyses [14] and basal and insulin-stimulated glucose uptake into isolated adipocytes were measured as previously described [26]. All study protocols were approved by the ethics committee of the University of Leipzig (Leipzig, Germany). All participants gave written informed consent before taking part in the study.

### Tagging SNP analysis in humans

From the ongoing Tübingen Family (TÜF) study for type 2 diabetes, healthy adults at increased risk for type 2 diabetes were recruited [27]. In the present study, a subset of 1896 participants with complete leukocyte and CRP measurements and a second subset of 420 subjects with complete IL6 measurements were selected for genotyping. Tagging SNPs covering the common genetic variation were identified and genotyping was conducted, as detailed in ESM Methods. Association analyses were performed by multiple linear regression analysis (least squares method) to adjust for the confounding variables sex, age and BMI. The study adhered to the Declaration of Helsinki, and its protocol was approved by the local ethics boards (Ethics Committees of the Eberhard Karls University Tübingen, Tübingen, Germany and the Karolinska Institute, Stockholm, Sweden). Informed written consent was obtained from all participants.

#### Statistical analysis

All data are reported as mean ± SEM. Statistical analysis was performed using Student’s *t* test for single comparisons and one-way ANOVA with Tukey’s post hoc test for differences between more than two groups. Differences between two groups over time were evaluated using a two-way ANOVA with Bonferroni’s or Sidak’s correction for multiple comparisons (GraphPad Prism version 6.00; GraphPad, La Jolla, CA, USA). For SNP analysis, JMP 11.2.0 (from SAS, Cary, NC, USA) was used for statistical analysis. Significance was accepted at *p* < 0.05, *p* < 0.01 and *p* < 0.001. Samples were randomised, and no data were omitted. The experimenters were not blind to group assignment.

## Results

### Overexpression of IFI202b increases body weight and fat mass

**Fig. 1:**
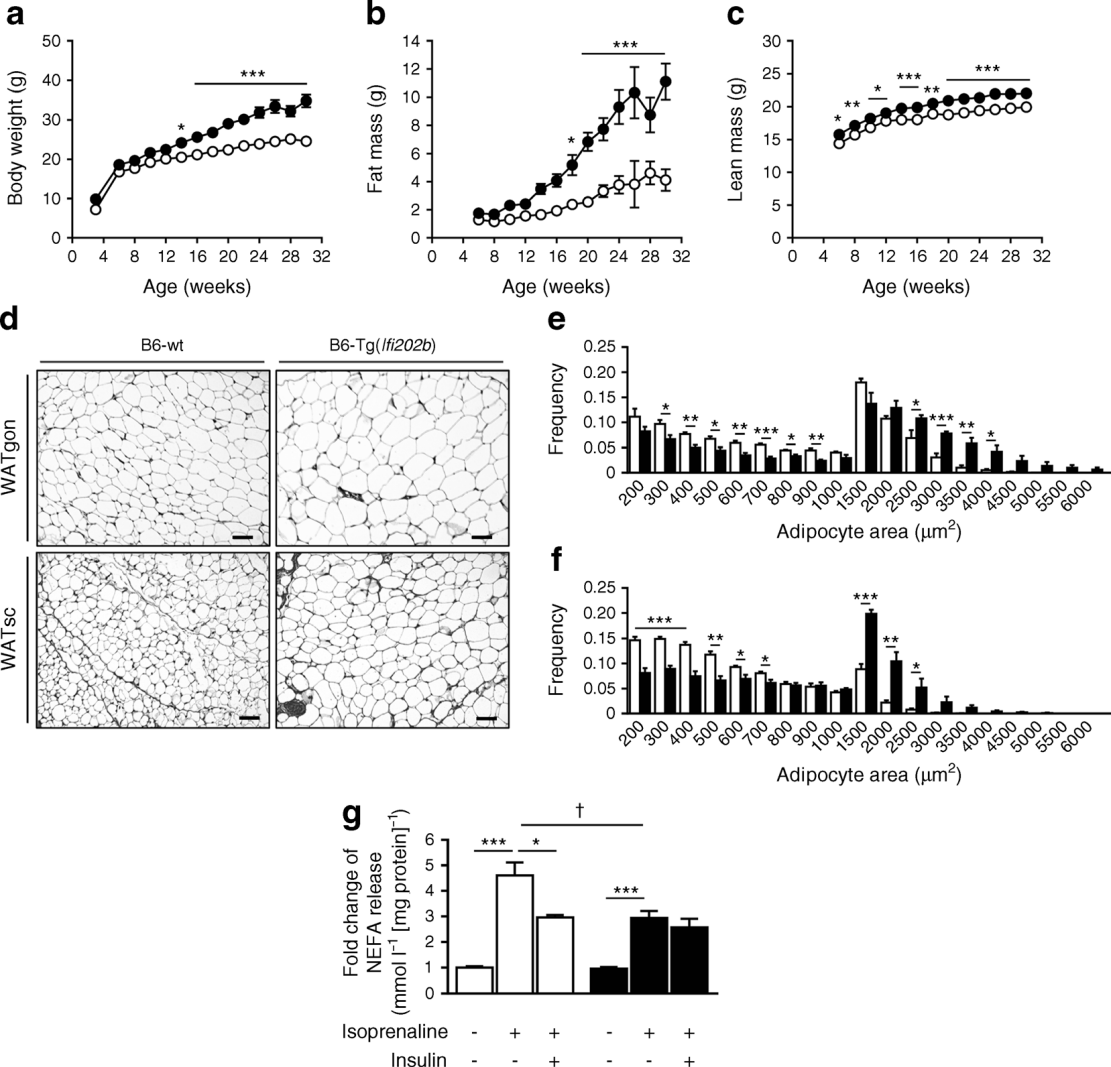
Overexpression of IFI202b increases body weight, fat mass and fat cell size, and affects lipolysis. c-Myc-*Ifi202b* cDNA was integrated into the ROSA26 locus of C57BL/6J mice. (**a**–**c**) B6-wt and B6-Tg(*Ifi202b*) mice were kept on an HFD and (**a**) body weight, (**b**) fat mass and (**c**) lean mass development were determined at indicated time points. B6-wt, *n* = 11; B6-Tg(*Ifi202b*), *n* = 13. *p < 0.05, **p < 0.01 and ***p < 0.001 (two-way ANOVA with Bonferroni’s multiple comparisons test). (**d**) Histological analyses of WATgon and WATsc from 8-week-old mice. Scale bar, 50 μm. (**e**, **f**) Respective frequencies of adipocyte areas in (**e**) WATgon and (**f**) WATsc. *n* = 5 mice per genotype. *p < 0.05, **p < 0.01 and ***p < 0.001, Student’s *t* test. (**g**) For the analysis of ex vivo lipolysis, WATgon explants of 8-week-old mice were stimulated with isoprenaline or isoprenaline plus insulin. Basal and conditioned media were collected after a 2 h incubation and NEFA levels were detected in the supernatant fractions. B6-wt, *n* = 5; B6-Tg(*Ifi202b*), *n* = 6. *p < 0.05 and ***p < 0.001 (comparison within groups); ^†^*p* < 0.05 (comparison between groups), Mann–Whitney test. Data are presented as mean ± SEM. White circles/bars, B6-wt; black circles/bars, B6-Tg(*Ifi202b*)

*Ifi202b* was identified as the most likely candidate responsible for the elevated body weight induced by the QTL *Nob3* recognised in female mice of an NZO × C57BL/6J F2 generation [14]. B6-wt mice do not express IFI202b as they lack the promoter and first exon [18]. Obese mice such as the NZO strain express particularly high levels of IFI202b in WAT, liver and muscle [14, 18]. To test whether IFI202b induces obesity in C57BL/6J mice, its c-Myc-tagged cDNA, fused to the ubiquitin C promoter, was integrated into the ROSA locus of the C57BL/6J mouse genome (ESM Fig. 1a). The resulting B6-Tg(*Ifi202b*) mice were fed a high-fat diet (HFD) and then characterised. At 8 weeks of age, *Ifi202b* expression was markedly elevated in all tissues of transgenic mice (ESM Fig. 1b), whereas mRNA levels were below the detection level (C_t_ value >35) in B6-wt mice. To compare c-Myc-IFI202b protein levels in transgenic mice with those in NZO mice, membrane/nuclear fractions of adipose tissue and liver samples were analysed by western blotting with anti-IFI202b and anti-c-Myc antibodies. In both NZO and B6-Tg(*Ifi202b*) mice, the adipose tissue contained more IFI202b than the liver. Furthermore, B6-Tg(*Ifi202b*) mice did not reach the tissue levels of IFI202b that were detected in NZO mice (ESM Fig. 1c). However, B6-Tg(*Ifi202b*) mice developed a higher body weight (Fig. 1a), fat mass (Fig. 1b) and lean mass (Fig. 1c) than B6-wt control mice. At the age of 30 weeks, the difference in body weight, fat mass and lean mass between the genotypes was 10.2 ± 1.9 g, 7.0 ± 1.6 g and 2.1 ± 0.5 g, respectively. These findings confirmed our recent assumption that IFI202b is responsible for the elevated body weight induced by the QTL *Nob3* [13, 14].

Histological analysis of WATgon and WATsc depots showed that adipocytes were larger in B6-Tg(*Ifi202b*) mice than in B6-wt mice (Fig. 1d). This was confirmed by quantitative analysis (Fig. 1e, f) and by the elevated leptin concentrations in B6-Tg(*Ifi202b*) mice at the age of 30 weeks (Table 1). In addition, B6-Tg(*Ifi202b*) mice displayed higher plasma triacylglycerol and NEFA concentrations than B6-wt mice at 6 weeks of age, an effect that was also visible at later time points (12 and 18 weeks; Table 1). To test whether lipolysis is affected in IFI202b-expressing mice, we used fat explants of 8-week-old mice to measure NEFA release under basal, isoprenaline-stimulated and isoprenaline plus insulin conditions. Basal lipolysis was comparable between both genotypes. Isoprenaline induced NEFA release from explants of B6-wt mice by about fivefold but from B6-Tg(*Ifi202b*)-derived explants this was only increased by about threefold (Fig. 1g). Notably, insulin did not sufficiently inhibit the stimulated lipolysis in explants of B6-Tg(*Ifi202b*) mice, indicating that IFI202b impairs insulin sensitivity.

**Table 1:** Plasma variables detected in B6-wt and B6-Tg(*Ifi202b*) mice at the post-absorptive state

Plasma variable	B6-wt	B6-Tg(*Ifi202b*)	*p* value
Triacylglycerol, mg/ml			
Week 6	383.90 ± 30.31 (*n* = 10)	511.78 ± 41.63 (*n* = 9)	0.022
Week 12	271.20 ± 31.78 (*n* = 10)	403.70 ± 47.75 (*n* = 9)	0.031
Week 18	228.80 ± 13.23 (*n* = 10)	463.89 ± 43.21 (*n* = 8)	<0.0001
NEFA, mmol/l			
Week 6	1.81 ± 0.11 (*n* = 10)	2.28 ± 0.12 (*n* = 9)	0.009
Week 12	1.21 ± 0.07 (*n* = 10)	1.57 ± 0.08 (*n* = 9)	0.005
Week 18	1.27 ± 0.05 (*n* = 10)	2.08 ± 0.08 (*n* = 8)	<0.0001
Leptin, ng/ml			
Week 12	2.91 ± 0.83 (*n* = 7)	5.77 ± 1.36 (*n* = 9)	0.139
Week 30	7.83 ± 1.50 (*n* = 10)	41.83 ± 5.81 (*n* = 12)	<0.0001

### IFI202b expression impairs insulin sensitivity and induces fatty liver

**Fig. 2:**
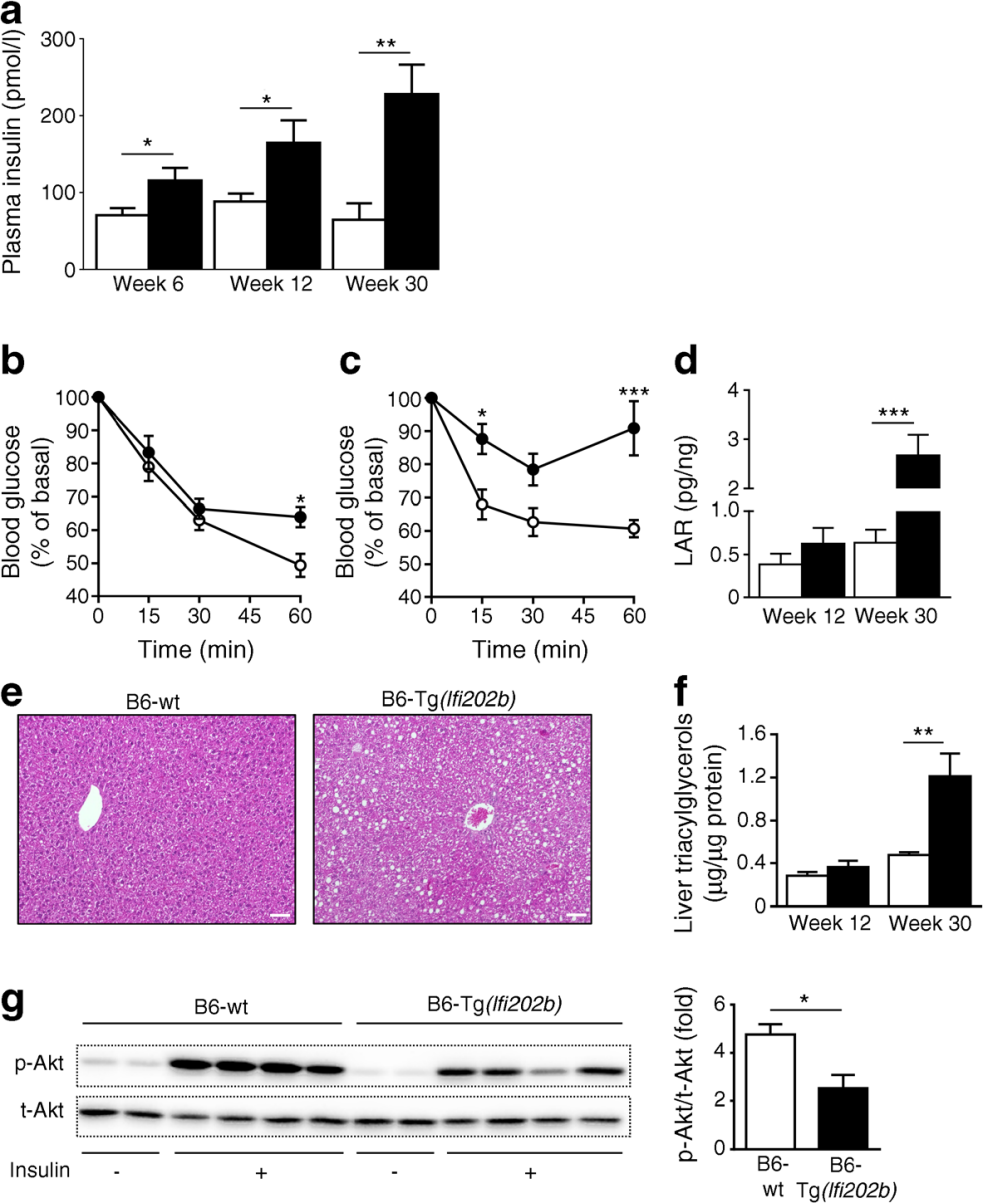
Overexpression of IFI202b induces insulin resistance. (**a**) Plasma insulin concentrations were measured in B6-wt and B6-Tg(*Ifi202b*) mice at the indicated time points. *n* = 9 mice per genotype. (**b**, **c**) ITTs were performed when mice were aged (**b**) 12 weeks (B6-wt, *n* = 6; B6-Tg(*Ifi202b*), *n* = 9) and (**c**) 20 weeks (B6-wt, *n* = 5; B6-Tg(*Ifi202b*), *n* = 4) and blood glucose levels were measured at the indicated time points. (**d**) The leptin-to-adiponectin ratio (LAR) was calculated at 12 (B6-wt, *n* = 6; B6-Tg(*Ifi202b*), *n* = 9) and 30 weeks (B6-wt, *n* = 11; B6-Tg(*Ifi202b*), *n* = 12) of age. (**e**) Representative liver H&E stains of 30-week-old B6-wt and B6-Tg(*Ifi202b*) mice. Scale bars, 50 μm. (**f**) Quantification of triacylglycerol levels in liver extracts of 12- (B6-wt, *n* = 9; B6-Tg(*Ifi202b*), *n* = 8) and 30-week-old mice (*n* = 11); *p* = 0.235 for difference between the groups at 12 weeks of age. (**g**) Twelve-week-old B6-wt and B6-Tg(*Ifi202b*) mice were fasted for 6 h and treated with NaCl or insulin (0.75 IU/kg body weight) for 20 min and then liver lysates were evaluated for total Akt (t-Akt) and p-Akt by western blot analysis. Representative blots and quantification are shown (*n* = 4). White circles/bars, B6-wt; black circles/bars, B6-Tg(*Ifi202b*). Data are presented as mean ± SEM. *p < 0.05, **p < 0.01 and ***p < 0.001 for B6-Tg(*Ifi202b*) vs B6-wt mice (two-way ANOVA with Sidak’s multiple comparisons test or Student’s *t* test)

To test whether B6-Tg(*Ifi202b*) mice are indeed insulin resistant, we determined plasma insulin levels and found that levels were already significantly elevated in B6-Tg(*Ifi202b*) mice by the age of 6 weeks (Fig. 2a). However, neither blood glucose levels nor glucose tolerance differed between the genotypes (ESM Fig. 2a, b, c). ITTs showed that B6-Tg(*Ifi202b*) mice had impaired insulin sensitivity at 12 (Fig. 2b) and 20 weeks (Fig. 2c) of age when compared with B6-wt control mice. The leptin-to-adiponectin ratio, as another measure of systemic insulin resistance [28], was elevated in 30-week-old B6-Tg(*Ifi202b*) mice and confirmed the impaired insulin sensitivity (Fig. 2d). An overflow of NEFA from adipose stores is known to result in ectopic lipid accumulation [29], which itself contributes to insulin resistance. As B6-Tg(*Ifi202b*) mice exhibited higher plasma NEFA concentrations than the B6-wt control mice (Table 1), we analysed liver histology. Livers of 30-week-old B6-Tg(*Ifi202b*) mice showed more and larger lipid droplets than those of B6-wt mice (Fig. 2e). Biochemical detection of hepatic triacylglycerol concentrations supported this observation; livers of 12-week-old mice displayed a non-significant increase in fat content (Fig. 2f). Nevertheless, at this stage insulin sensitivity was impaired in IFI202b-expressing mice. At the age of 12 weeks, B6-wt and B6-Tg(*Ifi202b*) mice were injected with NaCl or insulin and killed 20 min later to analyse insulin sensitivity. Figure 2g shows that insulin-stimulated Akt phosphorylation was markedly lower in livers of B6-Tg(*Ifi202b*) mice when compared with B6-wt littermates, whereas muscle insulin sensitivity was not affected (ESM Fig. 2d).

### IFI202b expression increases during early stages of adipocyte differentiation

*Ifi202b* mRNA levels were markedly increased during adipogenesis, reaching a maximum at 24 h after application of the differentiation cocktail and then decreasing over time, with the lowest levels in mature adipocytes (ESM Fig. 3a). As IFI202b is thought to function as a transcriptional modulator, we tested whether its subcellular localisation changes during the differentiation process. Nuclear and cytosolic fractions of confluent 3T3-L1 pre-adipocytes, as well as of cells at different time points after induction of differentiation, were isolated and endogenous IFI202b was detected by western blotting (ESM Fig. 3b). Shortly after induction of adipogenesis (24 h), the total amount of IFI202b increased, as did the quantity of IFI202b that appeared in the nuclear fraction (ESM Fig. 3b). This observation was also confirmed by immunocytochemistry. At 24 h, IFI202b was mostly present in the nucleus of the cells, whereas on day 3 it was mainly located in the cytosol (ESM Fig. 3c).

### Overexpression of IFI202b increases *Zfp423* mRNA levels in 3T3-L1 cells and in adipose tissue of B6-Tg(*Ifi202b*) mice

**Fig. 3:**
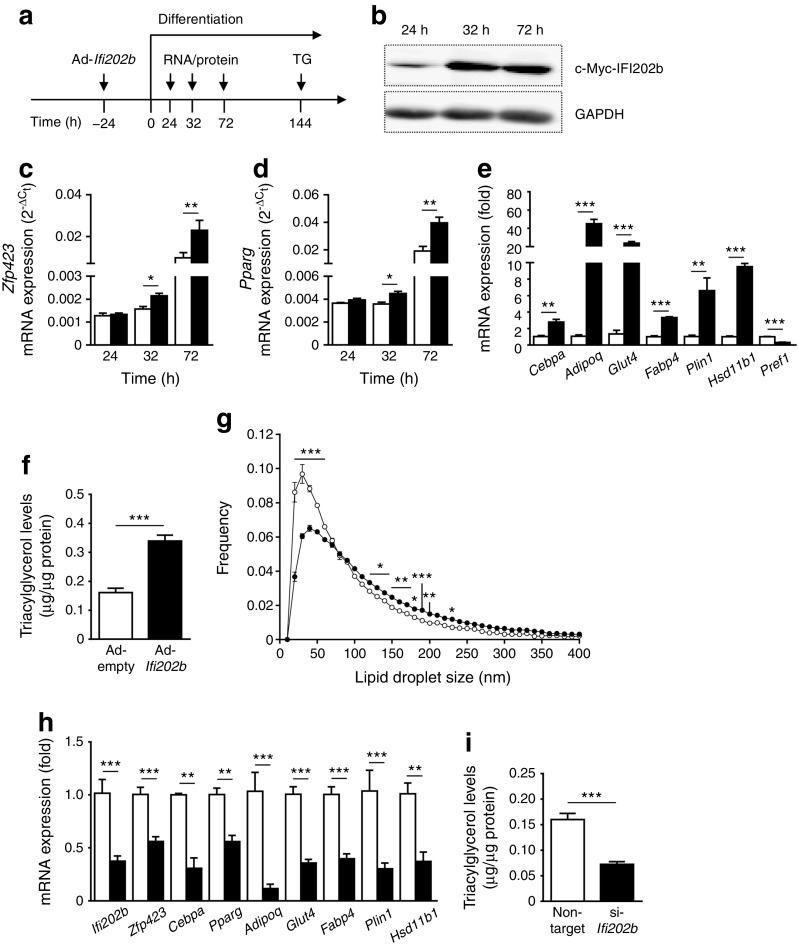
IFI202b overexpression induces *Zfp423* mRNA levels, accelerates adipogenesis and increases lipid storage in 3T3-L1 adipocytes. (**a**) Adenoviral-mediated overexpression of IFI202b (Ad-*Ifi202b*) was performed 24 h before a differentiation cocktail was applied. An empty adenovirus (Ad-empty) was used as a control. Cells were harvested at the indicated time points for quantitative real-time PCR, western blot and triacylglycerol (TG) analysis. (**b**) Overexpression of c-Myc-tagged IFI202b in 3T3-L1 cell lysates at the indicated time points. GAPDH was used as a loading control. (**c**–**e**) Gene expression levels of (**c**) *Zfp423* and (**d**) *Pparg* at indicated time points and of (**e**) adipocyte-specific genes (at 72 h) in control and IFI202b-overexpressing 3T3-L1 pre-adipocytes (non-target siRNA, *n* = 5; si-*Ifi202b*, *n* = 6). (**f**) Triacylglycerol content in virus-infected 3T3-L1 cells was measured at day 6 of differentiation (*n* = 12). (**g**) Frequency of lipid droplets at the indicated sizes in differentiated 3T3-L1 adipocytes after adenoviral-infection with Ad-empty or Ad-*Ifi202b* was detected by automated imaging acquisition (16 images per well, *n* = 5). (**c**–**g**) White bars/circles, Ad-empty; black bars/circles, Ad-*Ifi202b*. (**h**, **i**) IFI202b expression was suppressed by transfecting confluent 3T3-L1 cells using specific siRNA. One day after electroporation, differentiation was induced and cells were harvested (**h**) 2 days later for quantitative real-time PCR analysis of the indicated adipocyte marker genes and (**i**) 5 days later for detection of triacylglycerol levels (non-target siRNA, *n* = 5; si-*Ifi202b*, *n* = 6). (**h**–**i**) White bars, non-target siRNA; black bars, *Ifi202b* siRNA (si-*Ifi202b*). Data are presented as mean ± SEM. *p < 0.05, **p < 0.01 and ***p < 0.001, Student’s *t* test or two-way ANOVA with Sidak’s multiple comparisons test

The data above indicate that IFI202b is involved in early steps of adipogenesis. To identify downstream regulators, we analysed the WAT transcriptome of young B6-wt and B6-Tg(*Ifi202b*) mice. As our earlier studies found IFI202b to be highly expressed in WAT and nearly absent in BAT [14], a focus was put on transcription factors that are relevant for white fat cells. An important candidate that exhibited elevated expression in B6-Tg(*Ifi202b*) WAT was *Zfp423*, a multi-zinc finger transcription factor that is an essential determinant of pre-adipocyte commitment [30] and relevant for maintaining white adipocyte identity [31]. To determine whether *Zfp423* expression is induced by IFI202b, 3T3-L1 cells were infected by an *Ifi202b*-encoding adenovirus 1 day before the differentiation cocktail was applied (Fig. 3a). IFI202b protein was detected 24 h after induction of differentiation and further increased to stable levels at 32 and 72 h after differentiation (Fig. 3b). *Zfp423* showed a significantly higher expression level in IFI202b-overexpressing cells than in mock-infected cells, 32 h and 72 h after induction of differentiation (Fig. 3c). Since ZFP423 acts upstream of PPARγ [30], we also tested the effect of IFI202b on this key adipogenic regulator. As with *Zfp423*, the expression level of *Pparg* was significantly higher in IFI202b-overexpressing cells (Fig. 3d). We next analysed the expression of additional early and late adipogenic marker genes at 72 h. We confirmed our previous finding that *Hsd11b1* shows an elevated expression in response to IFI202b [14]. Several other genes (*Cebpa*, *Adipoq*, *Glut4*, *Fabp4* and *Plin1*) exhibited significantly higher mRNA levels in comparison with control cells (Fig. 3e) whereas mRNA levels of the pre-adipocyte marker *Pref1* were significantly reduced. Quantification of cell triacylglycerol levels at day 6 of differentiation indicated that IFI202b-overexpressing cells stored significantly more lipids (Fig. 3f) and exhibited a higher frequency of large lipid droplets than control cells (Fig. 3g). As expected, suppression of IFI202b in confluent 3T3-L1 pre-adipocytes resulted in reduced expression of *Zfp423* and other adipocyte-specific transcripts (Fig. 3h) and in a decline in triacylglycerol levels (Fig. 3i).

**Fig. 4:**
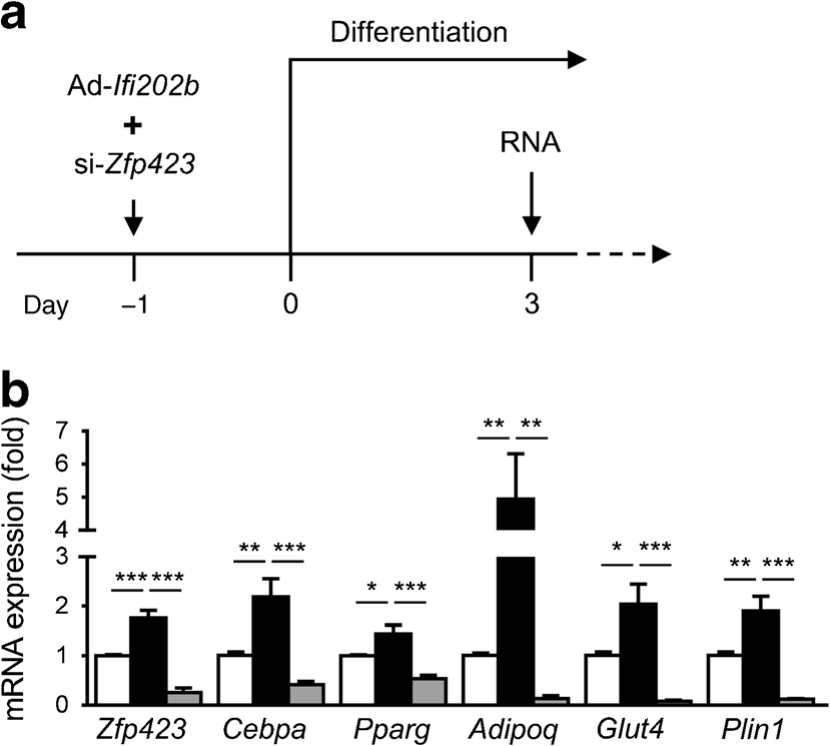
Suppression of ZFP423 expression abolishes the effects of IFI202b on adipogenesis in 3T3-L1 cells. (**a**) One day before the induction of differentiation, 3T3-L1 cells were treated with non-target siRNA or *Zfp423* siRNA (si-*Zfp423*) plus the *Ifi202b*-specific adenovirus (Ad-*Ifi202b*). (**b**) On day 3 of differentiation, mRNA was isolated for quantitative real-time PCR analysis of the indicated genes. White bars, empty adenovirus plus non-target siRNA; black bars, Ad-*Ifi202b* plus non-target siRNA; grey bars, Ad-*Ifi202b* plus si-*Zfp423*. Data are presented as mean ± SEM. *n* = 5. *p < 0.05, **p < 0.01 and ***p < 0.001 (one-way ANOVA with Tukey’s multiple comparisons test)

To assess whether the effects of IFI202b depend on the action of ZFP423, we inhibited ZFP423 expression in IFI202b-overexpressing 3T3-L1 cells (Fig. 4a). Transfection of cells with a *Zfp423*-specific siRNA significantly depleted *Zfp423* mRNA levels in IFI202b-overexpressing cells and this completely blocked the ability of IFI202b to induce adipogenesis (Fig. 4b), indicating that IFI202b mediates its action via ZFP423.

### Role of the human orthologue *IFI16* in SGBS adipocyte differentiation

**Fig. 5:**
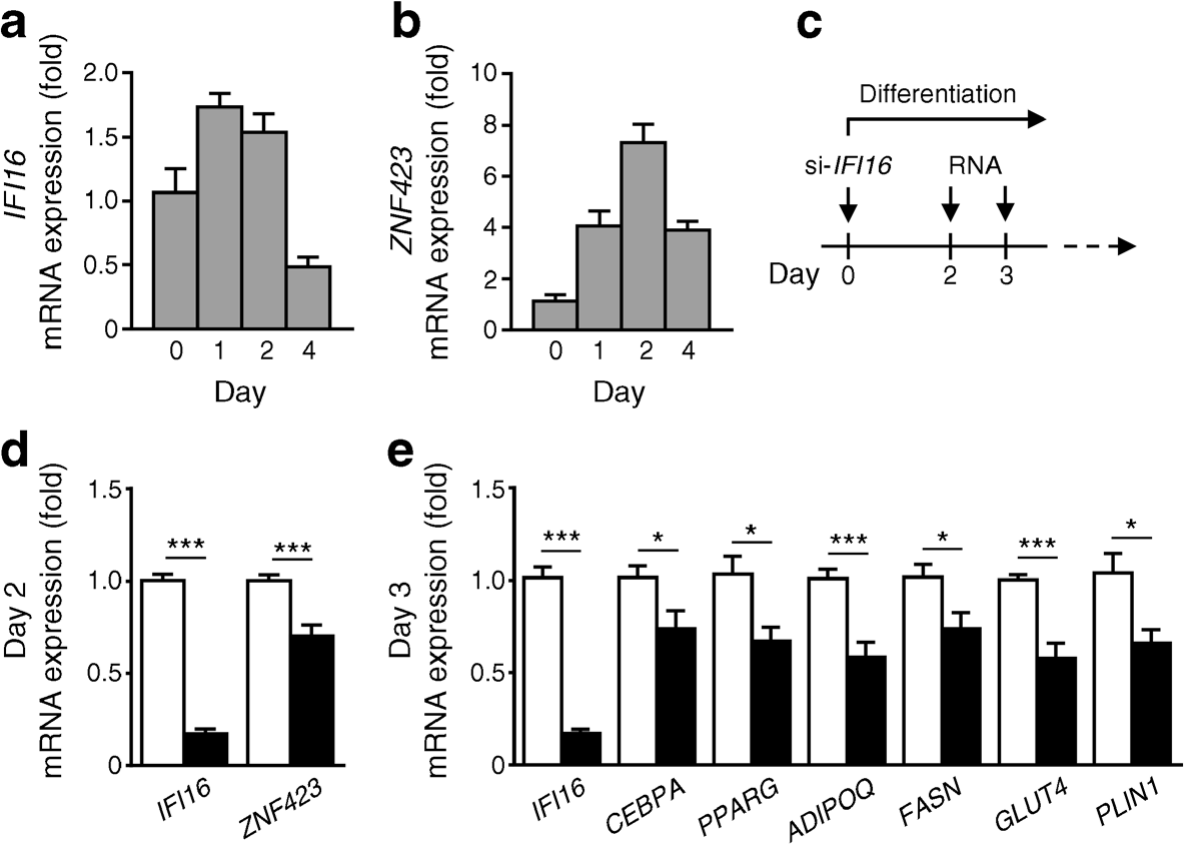
Suppression of IFI16 in human SGBS pre-adipocytes decreases *ZNF423* expression and adipocyte differentiation. (**a**) *IFI16* and (**b**) *ZNF423* mRNA levels in human SGBS pre-adipocytes at the indicated time points during early differentiation (*n* = 6). (**c**) Study design of SGBS cell experiments. (**d**, **e**) Quantitative real-time PCR analysis of indicated genes on (**d**) day 2 and (**e**) day 3 after IFI16 depletion via siRNA. White bars, non-target siRNA; black bars, *IFI16* siRNA (si-*IFI16*). Data are mean ± SEM (non-target si RNA, *n* = 9; si-*IFI16*, *n* = 8). *p < 0.05 and ***p < 0.001 (Student’s *t* test)

As *IFI16* is the most likely human orthologue of *Ifi202b* [14], and to translate our findings to humans, we examined the expression of *IFI16* and the *Zfp423* orthologue *ZNF423* during early differentiation of human SGBS cells. As observed with *Ifi202b* in 3T3-L1 cells, *IFI16* mRNA levels increased after induction of differentiation and subsequently decreased (Fig. 5a). Expression of *ZNF423* rose up to day 2 and dropped at day 4 of differentiation (Fig. 5b). Depletion of IFI16 expression in SGBS pre-adipocytes via specific siRNA (Fig. 5c) resulted in a significant reduction in *ZNF423* mRNA levels (Fig. 5d) and impaired adipocyte differentiation as reflected by lower levels of *CEBPA*, *PPARG*, *ADIPOQ*, *FASN*, *GLUT4* and *PLIN1* (Fig. 5e). Thus, like IfI202b in mice, IFI16 is involved in human adipogenesis.

### *Ifi202b* enhances the white adipocyte phenotype and inhibits the thermogenic gene program

**Fig. 6:**
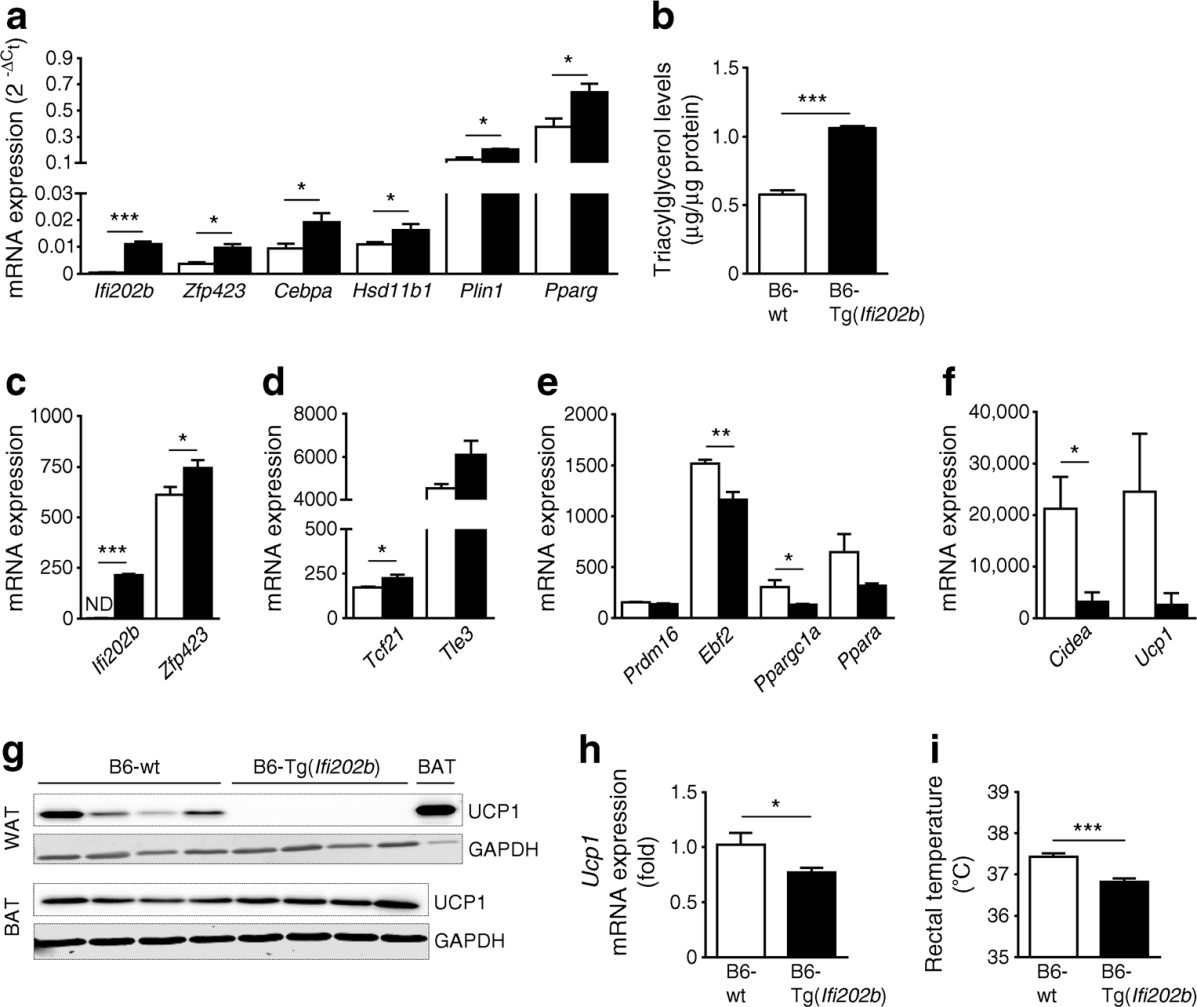
Overexpression of IfI202b in C57BL/6J mice increases *Zfp423* expression, promotes white adipocyte identity and reduces body temperature. (**a**) IfI202b was overexpressed in C57BL/6J mice and SCA1-positive adipogenic precursor cells were isolated from WATsc of 8-week-old mice on a standard diet. Cells were grown to confluence, treated with a specific differentiation cocktail up to day 12. Expression levels of indicated genes were analysed by quantitative real-time PCR. (**b**) Triacylglycerol content of mouse differentiated adipogenic precursor cells (*n* = 6 per genotype). (**c**–**f**) When comparing WATgon from B6-Tg(*Ifi202b*) mice vs B6-wt control mice (*n* = 4 mice per genotype), (**c**) *Zfp423* mRNA levels were significantly higher, (**d**) expression levels of white-selective genes were elevated and (**e**) mRNA levels of transcription factors and (**f**) marker genes of thermogenic brown fat determination were reduced. (**g**) Protein levels of UCP1 in WATgon (20 μg protein) and BAT (2 μg protein) of 8-week-old B6-wt and B6-Tg(*Ifi202b*) mice. GAPDH was used as a loading control. (**h**) *Ucp1* mRNA expression in BAT of 8-week-old mice. B6-wt, *n* = 5; B6-Tg(*Ifi202b*), *n* = 7. (**i**) Rectal temperature of 8-week-old mice. B6-wt, *n* = 8; B6-Tg(*Ifi202b*), *n* = 7. White bars, B6-wt; black bars, B6-Tg(*Ifi202b*). Data are expressed as mean ± SEM *p < 0.05, **p < 0.01 and ***p < 0.001 (Student’s *t* test). In (**c**–**f**), *Tle3*, *p* = 0.061; *Prdm16*, *p* = 0.106; *Ppara*, *p* = 0.116; *Ucp1*, *p* = 0.106. ND, not detected

To confirm the results obtained in cell culture experiments, SCA1-positive precursor cells were isolated from WATsc of young B6-wt and B6-Tg(*Ifi202b*) mice to determine their adipogenic potential. Precursor cells derived from B6-Tg(*Ifi202b*) mice exhibited a markedly elevated adipogenic potential as reflected by increased expression levels of *Zfp423*, *Cebpa*, *Pparg* and *Plin1* (Fig. 6a) as well as significantly increased triacylglycerol concentrations (Fig. 6b) in comparison with precursor cells isolated from of B6-wt mice.

Elevated *Zfp423* expression was also detected in WATgon of 8-week-old B6-Tg(*Ifi202b*) mice as compared with B6-wt mice (Fig. 6c). Recently, Shao and colleagues described ZFP423 as a critical regulator maintaining white adipocyte identity through the suppression of thermogenic genes, such as *Ebf2* and *Prdm16* [31]. We screened our transcriptome data for differentially expressed white-selective and thermogenic genes and detected higher mRNA levels of white marker genes (*Tcf21* and *Tle3*, Fig. 6d) and strikingly lower levels of beige-like marker genes (*Ebf2*, *Ppargc1a*, *Ppara*, *Cidea* and *Ucp1*, Fig. 6e, f), which also resulted in a stable downregulation of UCP1 (Fig. 6g). UCP1 was detectable in WATgon from B6-wt mice but absent in all samples from *Ifi202b* transgenic mice, supporting our hypothesis that IfI202b promotes the white adipocyte phenotype. In line with this, overexpression of IfI202b in WT-1 brown adipocytes resulted in a significant decrease of thermogenic genes including *Prdm16*, *Cidea* and *Ucp1* (ESM Fig. 4c–g). Even though we also detected lower expression levels of *Ucp1* mRNA in BAT from B6-Tg(*Ifi202b*) mice (Fig. 6h), this effect was not translated into protein levels (Fig. 6g).

Thus, the impaired browning capacity of WAT in *Ifi202b* transgenic mice might be responsible for the significantly lower body temperature of B6-Tg(*Ifi202b*) mice (Fig. 6i). Taken together, the increased expression of IfI202b induces *Zfp423* expression, resulting in a lack of beiging/browning of WAT, finally leading to a lower body temperature and obesity.

### Elevated expression of human *IFI16* associates with larger adipocytes and increased inflammatory cytokines

**Fig. 7:**
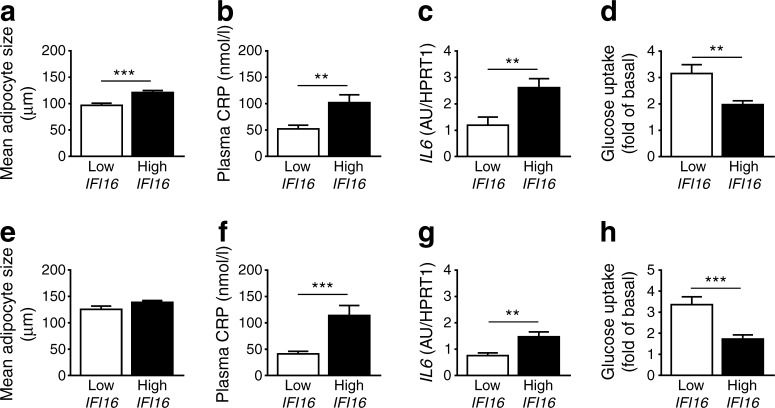
Higher human *IFI16* expression in adipose tissue is associated with larger adipocytes and increased levels of inflammatory markers. Of 166 obese individuals, the 10% with the highest and 10% with the lowest *IFI16* expression in the visceral (**a**–**d**) and subcutaneous fat depot (**e**–**h**) were selected. (**a**, **e**) Adipocyte size was measured, along with (**b**, **f**) plasma CRP levels, (**c**, **g**) *IL6* expression levels and (**d**, **h**) insulin-stimulated glucose uptake in isolated adipocytes. Data are presented as mean ± SEM. (**a**) *n* = 20; (**b**) *n* = 20; (**c**) *n* = 10 for low *IFI16* and *n* = 12 for high *IFI16*; (**d**) *n* = 18 for low *IFI16* and *n* = 20 for high *IFI16*; (**e**) *n* = 13 for low *IFI16* and *n* = 19 for high *IFI16*; (**f**) *n* = 20 for low *IFI16* and *n* = 19 for high *IFI16*; (**g**) *n* = 10 for low *IFI16* and *n* = 11 for high *IFI16*; (**h**) *n* = 17 for low *IFI16* and *n* = 16 for high *IFI16*. **p < 0.01 and ***p < 0.001 (Student’s *t* test). In (**e**), *p* = 0.059. AU, arbitrary units

*IFI16*, the human orthologue of *Ifi202b*, exhibits an elevated expression in visceral adipose tissue of obese as compared with lean individuals [14]. Here we assessed the *IFI16* expression in visceral and subcutaneous WAT of 166 obese individuals and analysed several variables of adipocyte function. The group of individuals with the 10% highest *IFI16* expression exhibited larger adipocytes in both fat depots than the individuals with the 10% lowest expression of *IFI16* (Fig. 7a), although this difference was not significant in subcutaneous fat (Fig. 7e). Concentration of plasma C-reactive protein (CRP) and *IL6* expression in fat depots were significantly higher in individuals with high *IFI16* levels (Fig. 7b, c, f, g), pointing towards an inflammatory response of adipose tissue under conditions of high *IFI16* expression. This was accompanied by a significantly reduced insulin-stimulated glucose uptake under conditions of high *IFI16* expression in both fat depots (Fig. 7d, h). Similarly, IFI202b overexpression in mice resulted in increased expression levels of inflammatory cytokines including *Il6*, *Tnfa* and *Ccl2* (ESM Fig. 5). We further screened tagging SNPs of *IFI16* for an association with inflammatory markers obtained in the TÜF study [27]. Interestingly, two of the rare *IFI16* alleles (CC allele of rs1417806 and AA allele of rs856077) were associated with elevated IL6 (*p* = 0.040) and CRP (*p* = 0.012) plasma levels, respectively. These data indicate that *IFI16* participates in elevated fat storage and adipose tissue inflammation.

## Discussion

Our data provide direct functional evidence for the role of *Ifi202b/IFI16* as an obesity gene and shows that it adjusts adipocyte commitment, maintenance of white adipocyte identity, fat cell size and the inflammatory state of adipose tissue. In 3T3-L1 and primary adipocyte progenitor cells, IfI202b induced *Zfp423* expression and thereby activated pro-adipogenic genes, enhanced whitening and inhibited the adipocyte thermogenic gene program. The consequent larger lipid droplets and elevated triacylglycerol accumulation are evident in IFI202b-overexpressing mice, which exhibited elevated fat mass, adipocyte hypertrophy and insulin resistance. Data from human studies revealed that individuals expressing high levels of *IFI16* in adipose tissue exhibited larger fat cells, elevated CRP plasma concentrations and *IL6* mRNA levels and impaired insulin-stimulated glucose uptake in adipose tissues than those with low *IFI16* expression.

As IFI202b is mainly expressed in WAT and nearly absent in BAT [14], we hypothesised that it induces pathways involved in adipocyte whitening. We identified ZFP423 as a downstream target of IfI202b and were able to block the IFI202b-specific effects by suppressing ZFP423. ZFP423 is a vital white adipocyte determination factor with dual roles. First, ZFP423 is a marker of committed pre-adipocytes and regulates *Pparg* levels, thereby inducing adipocyte differentiation [32]. Second, ZFP423 maintains white adipocyte identity via the suppression of thermogenic gene expression. Inactivation of ZFP423 in mature adipocytes leads to the white-to-beige conversion of WAT cells via induction of the brown fat cell determination factors *Prdm16* and *Ebf2* [31]. Besides *Zfp423*, we detected an upregulation of the relevant adipogenic marker genes *Cebpa*, *Pparg*, *Plin1* and *Hsd11b1* in 3T3-L1 pre-adipocytes and in primary precursor cells of B6-Tg(*Ifi202b*) mice, indicating that IFI202b improves the commitment and differentiation capacity of pre-adipocytes. The fact that thermogenic genes including *Ebf2*, *Cidea* and *Ucp1* were reduced in WATgon of B6-Tg(*Ifi202b*) mice indicates that IFI202b mediates the maintenance of white fat cell identity by suppressing thermogenic genes. This provides a possible explanation for the lower body temperature and subsequent development of obesity in B6-Tg(*Ifi202b*) mice. As seen with IFI202b in 3T3-L1 cells, inhibition of IFI16 in human SGBS pre-adipocytes suppressed the expression of adipogenic genes (*ZNF423* in particular).

Often, elevated food intake is a main driver for the development of obesity. Although we found that food intake was not significantly affected by IFI202b overexpression in young mice, it was significantly increased in 25-week-old B6-Tg(*Ifi202b*) mice as compared with B6-wt mice (ESM Fig. 6). Together with the lack of WAT beiging, this could have contributed to the elevated body weight.

Patil et al recently showed that the adipose tissue-specific overexpression of the transcription factor ID1 (inhibitor of differentiation 1) caused an HFD-induced obesity by diminishing *Prdm16*. ID1 interacts with early B cell factor 2 (EBF2), thereby suppressing its transcriptional activity and resulting in reduced expression of *Prdm16* [33]. PR domain containing 16 (PRDM16) and EBF2 are both required to determine beige/brown adipocyte cell fate [31]. Their genetic manipulation also affects body weight. Transgenic mice overexpressing the transcription factor EBF2 were protected from HFD-induced obesity since EBF2 robustly stimulated beige adipocyte development in the WAT of mice, even under conditions of thermoneutrality [34]. In addition, overexpression of *Prdm16* in mice increased their energy expenditure, limited weight gain and improved glucose tolerance on an HFD [35].

Several genes that are upregulated in response to IFI202b overexpression can participate in elevated fat storage. 11β-hydroxysteroid dehydrogenase type 1 (11β-HSD1), which we have previously identified as downstream target of IFI202b [14], might increase lipid content in adipose tissue by altering the local action of glucocorticoids [36]. Elevated GLUT4 could increase glucose uptake and consequently the fat content as has been shown for the adipocyte-specific GLUT4 transgenic mouse [37]. Perilipin 1 protects triacylglycerol from lipolysis [38, 39] and could thereby contribute to larger adipocytes. In fact, we detected lower lipolysis in response to isoprenaline in *Ifi202b* transgenic mice.

As hypertrophic adipocytes secrete more leptin and less adiponectin, the leptin-to-adiponectin ratio has been proposed as a useful measure of insulin resistance [28]. Indeed, this ratio was higher in *Ifi202b* transgenic mice than in B6-wt mice; glucose decrease in ITTs was impaired and insulin levels were not sufficient to suppress isoprenaline-stimulated lipolysis in fat explants of transgenic mice, indicating that IFI202b induces insulin resistance. Obesity associates with dyslipidaemia, which leads to hepatic accumulation of triacylglycerol and to an increased hepatic synthesis of VLDL [40]. IFI202b-overexpressing mice showed enhanced plasma NEFA and triacylglycerol levels, with resulting ectopic hepatic lipid storage. We believe that an elevated action of IFI202b/IFI16 leads to hypertrophic adipocytes, elevated fat storage, induction of inflammatory cytokine expression and finally insulin resistance and fatty liver. Similar to our observations made in B6-Tg(*Ifi202b*) mice, elevated plasma triacylglycerol levels are often detected in obese individuals and adipose tissue dysfunction appears to be responsible for these abnormalities in lipid metabolism [41]. Pathological expansion of the adipose tissue results in inflammation and increased immune cell and macrophage infiltration, proposed as one mechanism to explain the development of insulin resistance [42]. In fact, humans with high *IFI16* mRNA levels displayed higher concentrations of CRP in the plasma and *IL6* expression in adipose tissue, effects that are associated with a lower insulin-stimulated glucose uptake.

Thus, IFI proteins could represent possible targets for altering adipogenesis and the development of obesity. Nevertheless, follow-up studies are needed to unravel the specific regulation and putative binding partners of IFI202b and IFI16.

In conclusion, our data show that IFI202b and its human orthologue IFI16 are key players in the development of obesity and insulin resistance: (1) by modulating fat storage in adipose tissue and liver; (2) by inhibiting beiging of WAT; and (3) by inducing inflammatory markers.

## Electronic supplementary material

ESM (PDF 379 kb)

## Data Availability

Generated and analysed datasets of this study are available from the corresponding author on reasonable request.

## Abbreviations

B6-wt: C57BL/6J wild-type
BAT: Brown adipose tissue
C/EBP: CCAAT/enhancer-binding protein
CRP: C-reactive protein
EBF2: Early B cell factor 2
HFD: High-fat diet
IFI: IFN-inducible protein
*Nob3*: New Zealand Obese obesity 3 (quantitative trait locus)
NZO: New Zealand Obese
PPARγ: Peroxisome proliferator-activated receptor γ
QTL: Quantitative trait locus
SCA1: Stem cell antigen 1
SGBS: Simpson–Golabi–Behmel syndrome
TÜF: Tübingen Family (study)
UCP1: Uncoupling protein 1
WAT: White adipose tissue
WATgon: Gonadal white adipose tissue
WATsc: Subcutaneous white adipose tissue
ZFP: Zinc finger protein
