# Supplementary material for: Increased Ifi202b/IFI16 expression stimulates adipogenesis in mice and humans
Source: Diabetologia. 2018 Feb 24;61(5):1167–79. doi: 10.1007/s00125-018-4571-9 (PMC6448999; doi:10.1007/s00125-018-4571-9)
Supplement: Supplementary file 1 — (PDF 379 kb) [file 125_2018_4571_MOESM1_ESM.pdf]

ESM Methods

Animals Mice were kept at a temperature of 20 ± 2°C with a 12:12 hours light-dark cycle and had ad libitum access to drinking water and to a high-fat diet containing 45 energy% from fat, 35 energy% from carbohydrates and 20 energy% from protein (D12451, Research Diets, Inc., New Brunswick, USA). All animal experiments were approved by the ethics committee of the State Office of Environment, Health and Consumer Protection (Federal State of Brandenburg, Germany).

Histological analysis Paraffin sections (2 µm) of WATsc, WATgon, BAT, and livers of B6-wt and B6-Tg(*Ifi202b*) mice were stained with hematoxylin and eosin. Microscopic images were captured with the Keyence BZ-9000 fluorescent microscope and the corresponding BZ-II Analyzer software (Keyence International, Mechelen, Belgium). Adipocyte areas of three histological images per mouse were defined using the Wimasis WimAdipose Image Analysis system (Wimasis, München, Germany).

Cell culture and adipocyte differentiation Mycoplasma-free 3T3-L1 cells (ATTC CL-173) were maintained at 37°C and 5% CO_2 in IMDM (PAA Laboratories, Pasching, Austria) supplemented with 10% newborn calf serum. Differentiation of confluent cells was induced by IMDM/10% FBS (PAN-Biotech) containing 3.3 µmol/l insulin (Roche), 0.5 mmol/l 3-isobutyl-1-methylxanthine (IBMX, Sigma-Aldrich) and 2 µmol/l rosiglitazone (Sigma-Aldrich). At day 3 and day 4, medium was replaced with IMDM (10% FBS, 3.3 µmol/l insulin) followed by IMDM/10% FBS at day 5 and day 6.

Human SGBS cells (mycoplasma test: negative) were provided from Dr. Wabitsch’s laboratory (University of Ulm, Medical Faculty, Ulm, Germany) and grown and differentiated as described in [1, 2].

Overexpression of IFI202b and analysis of lipid droplet formation in 3T3-L1 pre-adipocytes Confluent 3T3-L1 pre-adipocytes were infected with a c-Myc-*Ifi202b* encoding

adenovirus (VQAd *Ifi202b*, ViraQuest, Inc., North Liberty, USA) with MOI of 550. The adenovirus was preincubated in serum-free IMDM plus 0.5% poly-L-lysine hydrobromide (Sigma-Aldrich) for 100 min at RT and applied to cells in IMDM/10% NCS overnight. Empty adenovirus (VQAd EMPTY-2.6del) was used as control.

Lipid droplet formation in IFI202b overexpressing 3T3-L1 adipocytes was assessed by fluorescent staining (BODIPY for lipid droplets, Hoechst for nuclei and Syto60 for cytosolic staining, Invitrogen, Carlsbad, CA, USA) with automated image acquisition and analyzed as previously described [3].

RNA extraction and expression analysis Total RNA from cells was isolated with TRIzol Reagent (Thermo Fisher Scientific, Waltham, MA, USA) according to the manufacturer's instructions. Extraction of total RNA from mouse tissues was performed as described before [4]. RNA samples were reverse transcribed using the Moloney Murine Leukemia Virus Reverse Transcriptase (M-MLV RT, Promega Corporation, Madison, USA). Expression levels of the genes *Ucp1*, *Prdm16* and *Cidea* were detected using specific TaqMan Gene Expression Assays (Thermo Fisher Scientific).

All data were normalized referring to Livak and Schmittgen [5]. Actin beta (*Actb*) and eukaryotic translation elongation factor 2 (*Eef2*) expression was used as internal control for cells and mouse tissues, respectively. Gene expression profiling of WATgon of 8-week-old mice was performed using SurePrint G3 Mouse Gene Expression 8x60K Microarrays from Agilent Technologies (Santa Clara, CA, USA).

Tagging SNP analysis in human subjects The Tübingen Family (TÜF) study for type 2 diabetes recruited healthy adults at increased risk for type 2 diabetes (inclusion criteria: BMI ≥ 27, family history of diabetes, impaired fasting glucose, and/or previous gestational diabetes) [6]. Tagging SNPs (minor allele frequency > 0.1) were identified by Haploview tagger freeware (https://www.broadinstitute.org) based on genetic linkage data from the 1000

Genomes Project (http://browser.1000genomes.org; CEU population). For genotyping, mass spectrometry with the MassARRAY platform and iPLEX software from Sequenom (Hamburg, Germany) was applied. Due to the hypothesis-driven nature of the analyses, p values < 0.05 were considered statistically significant.

ESM References

1. Wabitsch M, Brenner REE, Melzner I, et al (2001) Characterization of a human preadipocyte cell strain with high capacity for adipose differentiation. Int J Obes 25:8–15.
2. Fischer-Posovszky P, Newell FS, Wabitsch M, Tornqvist HE (2008) Human SGBS cells - A unique tool for studies of human fat cell biology. Obes Facts 1:184–189.
3. Meissburger B, Ukropec J, Roeder E, et al (2011) Adipogenesis and insulin sensitivity in obesity are regulated by retinoid-related orphan receptor gamma. EMBO Mol Med 3:637–51.
4. Vogel H, Scherneck S, Kanzleiter T, et al (2012) Loss of function of Ifi202b by a microdeletion on chromosome 1 of C57BL/6J mice suppresses 11β-hydroxysteroid dehydrogenase type 1 expression and development of obesity. Hum Mol Genet 21:3845–57.
5. Livak KJ, Schmittgen TD (2001) Analysis of relative gene expression data using real-time quantitative PCR and. Methods 25:402–408.
6. Staiger H, Böhm A, Scheler M, et al (2013) Common Genetic Variation in the Human FNDC5 Locus, Encoding the Novel Muscle-Derived “Browning” Factor Irisin, Determines Insulin Sensitivity. PLoS One.

Electronic supplementary material (ESM)

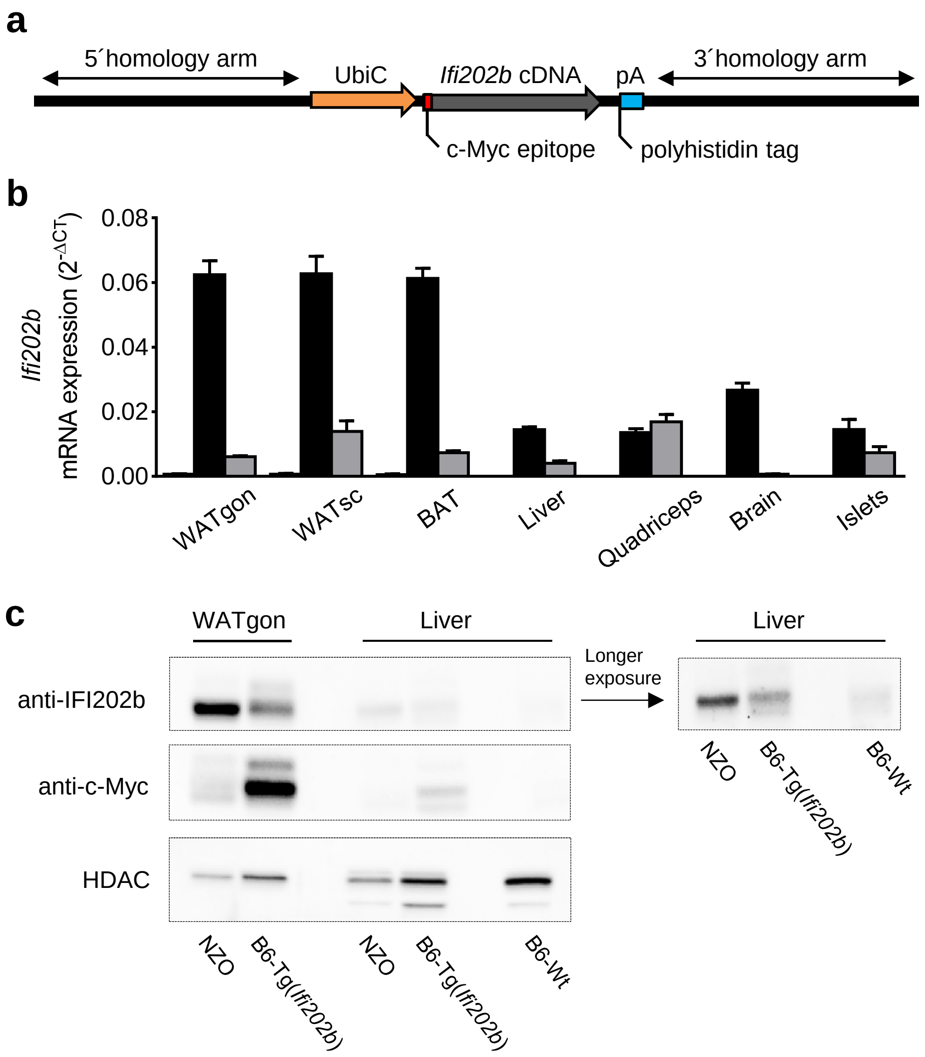

ESM Fig. 1 Expression of IFI202b in B6-wt, B6-Tg(*Ifi202b*) and NZO mice. (**a**) Structure of the targeting vector integrated into the ROSA26 locus of B6 mice in order to overexpress IFI202b. *Ifi202b* cDNA tagged with a c-Myc epitope at the N-terminus was fused to the ubiquitin C promoter and flanked by sequences corresponding with the ROSA locus. (**b**) *Ifi202b* mRNA levels in different tissues were detected by quantitative real-time PCR. Expression of *Ifi202b* was significantly higher in all tissues of B6-Tg(*Ifi202b*) mice than in B6-wt mice (one-way ANOVA with Tukey's *post hoc* test). White bars, B6-wt (n = 6); black bars, B6-Tg(*Ifi202b*) (n = 6); grey bars, New Zealand Obese (NZO, n = 5). Data are expressed as means ± SEM. (**c**) Analysis of c-Myc-IFI202b protein levels in membrane/nuclear fractions of gonadal white adipose tissue and livers of B6-wt, B6-Tg(*Ifi202b*) and NZO mice by western blotting with antibodies against IFI202b and c-Myc. HDAC was displayed as loading control.

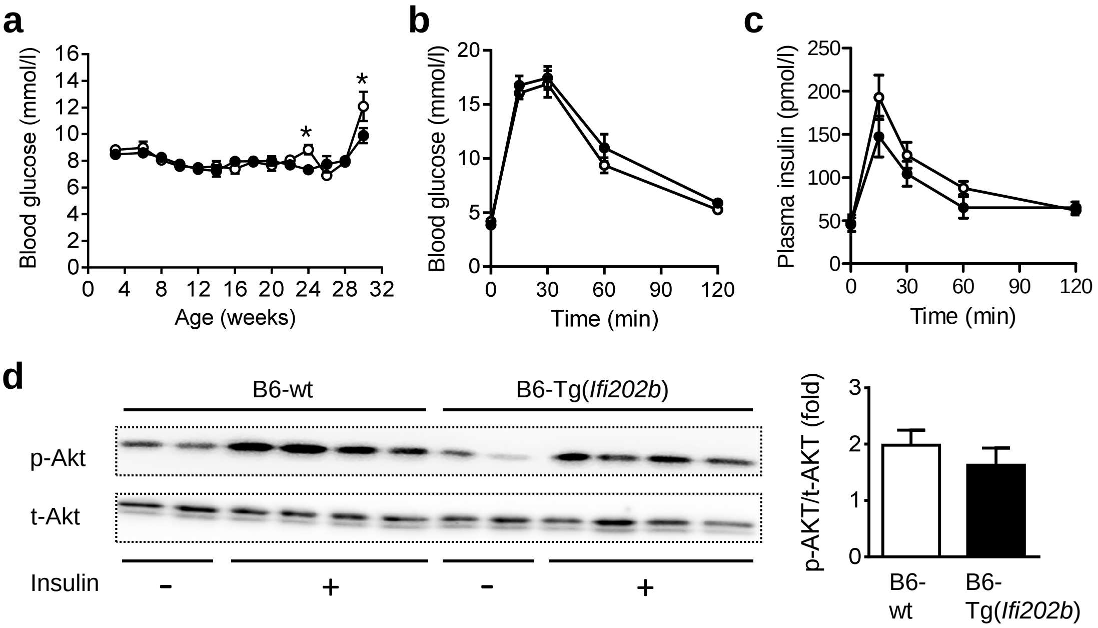

ESM Fig. 2 IFI202b expression does not increase blood glucose concentrations. (**a**) Blood glucose levels were measured every other week up to the age of 30 weeks (*n* = 10 mice per genotype). For an OGTT, 12-week-old B6-wt and B6-Tg(*Ifi202b*) mice were fasted overnight (2 g/kg body weight glucose by oral gavage) and (**b**) blood glucose and respective (**c**) plasma insulin levels were detected at the indicated time points (*n* = 9 mice per genotype). (**d**) Muscle lysates of 12-week-old B6-wt and B6-Tg(*Ifi202b*) mice that were fasted for 6 h and treated with NaCl or 0.75 IU/kg body weight insulin for 20 min were evaluated for total Akt (t-Akt) and p-Akt by western blotting. The right panel shows the quantification for insulin stimulated samples (*n* = 4). White circles, B6-wt; black circles, B6-Tg(*Ifi202b*). Data are presented as mean ± SEM. *$p < 0.05$ by two-way ANOVA with Bonferroni's correction for multiple comparisons.

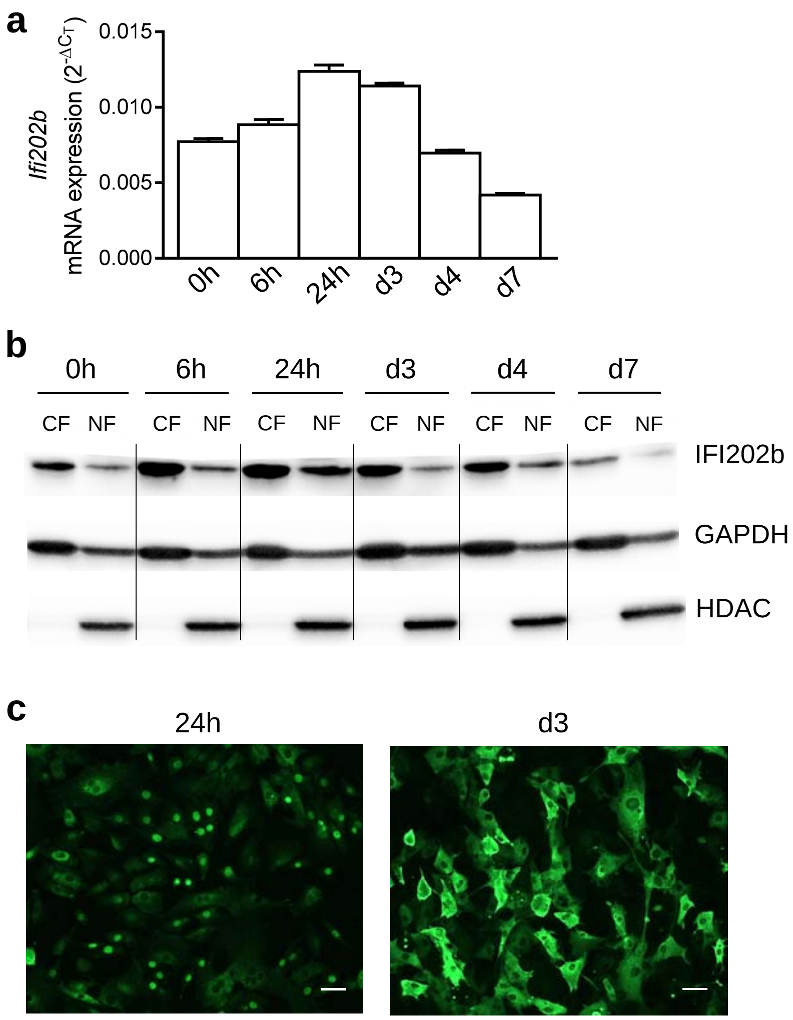

ESM Fig. 3 Shortly after induction of differentiation, IFI202b expression elevates which associates with a higher abundance of the protein in the nucleus. (**a**) Endogenous mRNA expression of *Ifi202b* was studied at the indicated time points during the differentiation of 3T3-L1 cells by quantitative real-time PCR. (**b**) Determination of the subcellular localization of IFI202b at the indicated time points of differentiation by western blotting and (**c**) by immunocytochemistry. Scale bar, 50 µm. CF, cytosolic fraction; NF, nuclear fraction

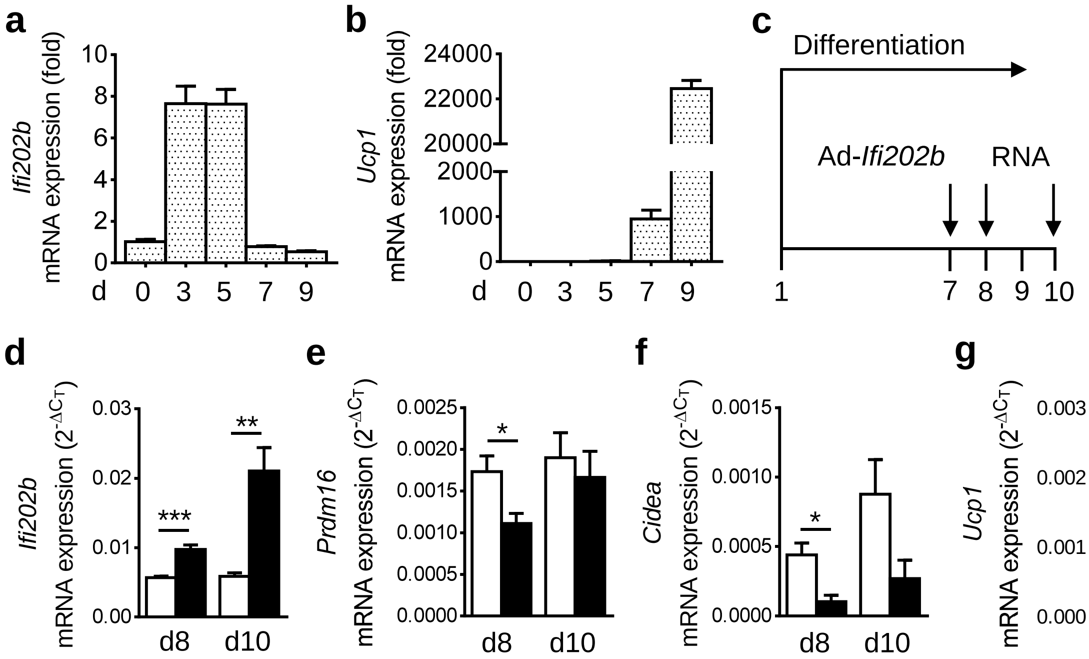

ESM Fig. 4 IFI202b overexpression in WT-1 brown adipocytes leads to reduced *Ucp1* expression levels. (**a**) Endogenous *Ifi202b* and (**b**) *Ucp1* mRNA levels in WT-1 cells at the indicated time points during brown adipocyte differentiation. (**c**) At day seven of differentiation, adenoviral-mediated overexpression of IFI202b was achieved and cells were harvested one and three days later for RNA isolation. (**d**) Transcript levels of *Ifi202b*, (**e**) *Prdm16*, (**f**) *Cidea* and (**g**) *Ucp1* in WT-1 cells treated with either empty or a *Ifi202b*-specific adenovirus at the indicated time points. White bars, empty adenovirus; black bars, *Ifi202b* adenovirus. Data are presented as mean ± SEM (n = 4). *$p < 0.05$, **$p < 0.01$, ***$p < 0.001$ by Student's t test. *Cidea* (d10), p = 0.074

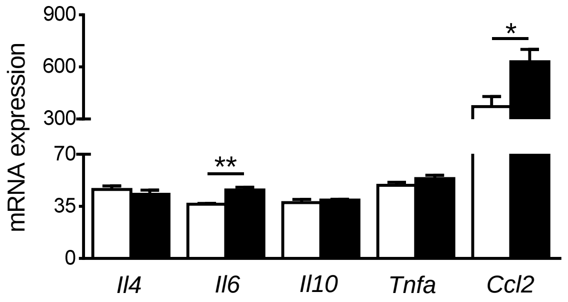

ESM Fig. 5 IFI202b expression in C57BL/6J mice alters the expression of inflammatory transcripts. Expression levels of indicated genes in gonadal white adipose tissue samples of 8-week-old B6-wt and B6-Tg(*Ifi202b*) mice (n = 4 mice/group). Data are presented as mean ± SEM. White bars, B6-wt; black bars, B6-Tg(*Ifi202b*). *$p < 0.05$, **$p < 0.01$ by Student's t test. *Tnfa*, $p = 0.182$

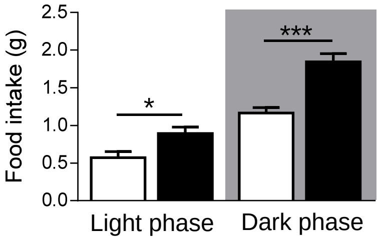

ESM Fig. 6 IFI202b overexpression in B657BL/6J mice resulted in a significant elevation of food intake as compared with B6-wt mice at the age of 25 weeks. Data are presented as mean ± SEM. White bars, B6-wt, $n = 12$; black bars, B6-Tg(*Ifi202b*), $n = 11$. *$p < 0.05$, ***$p < 0.001$ by Student's *t* test
